# Experimental Validation of Methods for Prophylaxis against Deep Venous Thrombosis: A Review and Proposal

**DOI:** 10.1155/2012/156397

**Published:** 2012-04-10

**Authors:** Paul S. Agutter, P. Colm Malone, Ian A. Silver

**Affiliations:** ^1^Theoretical Medicine and Biology Group, 26 Castle Hill, Glossop, Derbyshire, UK; ^2^Department of Pathology, University of Birmingham, Edgbaston, Birmingham B15 2TT, UK; ^3^Centre for Comparative and Clinical Anatomy, University of Bristol, Southwell Street, Bristol BS2 8EJ, UK

## Abstract

The experimental procedure by which the valve cusp hypoxia (VCH) hypothesis of the etiology of deep venous thrombosis (DVT) was confirmed lends itself to testing of methods of prophylaxis. Similar animal experiments could end the present exclusive reliance on statistical analysis of data from large patient cohorts to evaluate prophylactic regimes. The reduction of need for such (usually retrospective) analyses could enable rationally-based clinical trials of prophylactic methods to be conducted more rapidly, and the success of such trials would lead to decreased incidences of DVT-related mortality and morbidity. This paper reviews the VCH hypothesis (“VCH thesis”, following its corroboration) and its implications for understanding DVT and its sequelae, and outlines the experimental protocol for testing prophylactic methods. The advantages and limitations of the protocol are briefly discussed.

## 1. Introduction

Deep venous thrombosis (DVT) is a major concern for the management of hospital patients and a risk for the general population because of its high incidence. Its sequelae often lead to mortality (via pulmonary embolism (PE)) or chronic morbidity (postthrombotic syndrome), so prophylactic measures have long been considered priorities and are the foci of recent discussions and guidelines published on both sides of the Atlantic [1–4]. These and many similar papers rely on statistical analysis of data from large patient cohorts to evaluate prophylactic regimens. They mostly emphasize pharmacological anticoagulation (disabling of normal hemostatic function), with mechanical methods such as intermittent pneumatic compression assigned a secondary or adjunctive role. For example, on the basis of a review of randomized trials since 1950, the 2011 American College of Physicians guideline recommended (i) assessment of medical (including stroke) patients for the risk of thromboembolism, (ii) pharmacological prophylaxis with heparin or related drug unless there is a serious risk of bleeding, and (iii) mechanical prophylaxis with graduated compression stockings in the event of a bleeding risk [4].

However, despite continual refinements of the pharmaceutical approach, DVT/PE-related mortality has shown only minimal reduction during recent decades, as reviewed in, for example, [5, 6]. The incidence of secondary (hospital-acquired) DVT in the USA more than tripled during the period 1989–2006, and there was a 2.5-fold increase in the number of admissions with a primary diagnosis of PE [7]. Several recent studies have cast doubt on the prophylactic value of anticoagulants [4, 6, 8]. A comprehensive review of trials indicated that heparin did not reduce overall mortality, though it decreased the incidence of PE [4], and a large trial showed that anticoagulant prophylaxis is associated with a risk of serious and potentially fatal bleeding and little benefit in terms of DVT/PE prophylaxis [8]. Indeed, it has been suspected for more than half a century that heparin does not prevent the *initiation* of DVT. In 1952, for instance, summarizing the results of a series of experiments in which intravenous fibrogenesis was induced in dogs, Samuels and Webster wrote “It appears, from these experiments, that fibrin may appear on the injured vessel walls even in cases where the blood is rendered incoagulable by heparin” [9]. Nevertheless, anticoagulant prophylaxis remains the standard of care for patients considered at risk for DVT and its sequelae.

Two connected reasons can be suggested for this apparent disjunction between current practice and accumulating evidence. First, since blood coagulation occurs at some stage during thrombogenesis (venous or otherwise), logic suggests that anticoagulants should inhibit the process. Data at odds with that logic are apt to be ignored because they challenge established belief as well as practice. However, it now seems likely that “coagulation” is not the primary event in the generation of a venous thrombus, but rather a consequence of the morbid pathological process within one or more valve pockets that can lead to thrombogenesis [10, 11]. The incipient conflict in the recent literature could be resolved if this account of the etiology of DVT, the *valve cusp hypoxia (VCH) *thesis, were more widely appreciated. This thesis [10, 11] is summarized in the following sections and its implications are explored. Second, the crucial evidence confirming the VCH thesis was obtained from an animal experiment [12] that lends itself to testing approaches to prophylaxis. The results of further studies using this experimental procedure should answer the question “Is such-and-such a prophylactic measure effective?” and end the current reliance on statistical analysis of large bodies of patient data. In this paper we will describe this approach to the validation of prophylactic measures. It involves a reproducible, noninvasive method for inducing morphologically accurate, autochthonous deep venous thrombi in an animal model [12].

The paper is structured as follows. In Section 2 we summarize the VCH thesis, and in Section 3 we review some of its implications.  Section 4 comprises a description of our proposed experimental protocol. The advantages and limitations of this approach are discussed in Section 5, and conclusions are drawn.

## 2. The Etiology of DVT: The VCH Thesis

Venous blood flow is influenced by two main factors: *vis a tergo* resulting from the continuous entry of blood from the capillary beds into the venules and mechanical pumping resulting, for example, from upward pressure on the soles of the feet during walking and from contractions of the gastrocnemius and other skeletal muscles. When there is little or no mechanical pumping, *vis a tergo* dominates and the flow is *continuous* (laminar, streamline, nonpulsatile); when mechanical pumping takes place, the flow is *discontinuous* (intermittent, pulsatile). According to the VCH thesis [10, 11], DVT may follow when pathologically long periods (over 1.5–3.0 hours) of continuous venous blood flow result in severe hypoxemia in one or more valve pockets, leading in turn to injurious hypoxia of the valve cusp endothelium in the depths of the pocket(s) [11]. Should a burst of discontinuous flow, however brief, flush out the stagnant blood from such oxygen-starved pocket(s), the next refill of the pocket will automatically carry fresh, normally oxygenated blood from the vein lumen, containing living leukocytes and platelets. Such newly introduced cells may instantly marginate, settle on, and attack the lately unperfused, moribund endothelium. Living phagocytes are therefore likely to attach themselves to the dying parietalis endothelial layer lining the inner aspect of the valve cusp. Alternating protracted periods of such continuous (i.e., streamline) blood flow with restored normal (i.e., discontinuous) flow can progressively build up a burgeoning thrombus comprising accumulated dead blood cells and concomitantly generated fibrin.

In more detail, the pathogenic process can be envisaged in terms of the following steps.

- Normal venous blood flow is associated with a four-phase valve cycle, which was analyzed in detail by Lurie and colleagues [13, 14]. This cycle is strongly dependent on the pulsatility of flow, which in the lower limbs is ensured, for example, by the calf muscle pump and the upward pressure on the soles of the feet during walking. In simple terms, the valve cusp leaflets are forced apart (opening the valve) when the pressure caudad exceeds the pressure cephalad and are forced together again (closing the valve) when the converse holds. Under conditions when the calf muscle pump does not operate and there is no upward pressure on the soles of the feet, venous blood flow depends mostly on *vis a tergo *and becomes continuous (streamline), so the four-phase cycle ceases to operate. The valve cusp leaflets remain partly open, partly closed, their tips oscillating slightly in the streamline flow owing to the Venturi effect.
- Under these conditions, the blood within the valve pockets scarcely exchanges with the luminal blood in the vein. Instead, it circulates within the pocket, describing two vortices: a primary vortex near the mouth of the pocket, driven by the passing luminal blood and the Venturi effect on the cusp leaflet, and a slow secondary vortex in the depths of the pocket, driven by the primary vortex [15–17].
- Respiring blood cells in this secondary vortex, and the endothelial cells in contact with it, progressively deplete the oxygen store of the blood trapped in such pockets. Theoretical calculations predict [11], and experimental measurements confirm [18], that the hypoxemia can become sufficiently severe after 1.5–3.0 hours to injure the endothelial cells; the PO_2_ registered in the cells deep in the pocket fell below the detection limit of the oxygen electrode (<0.14 kPa) after such a period [18]. (Since the blood flowing in the vein lumen remains oxygenated, this strongly suggests that the valve cusp leaflet is effectively impermeable to oxygen.)
- Although the endothelial cells in the depths of the valve pocket can maintain themselves temporarily by anaerobic metabolism [19], they are inevitably affected by this sustained and extreme hypoxia. In particular, the cells on the inner (parietalis) surface of the valve cusp leaflet are likely to become necrotic because—unlike the vein wall endothelial layer—they have no blood supply from vasa venarum [20–23]. In contrast, the endothelial cells on the circumferential wall of the valve pocket may survive because they receive a vasa venarum supply [20, 21] and hence are less susceptible to oxygen depletion of the blood in the pocket. Therefore, while they may become hypoxic, they need not become fatally anoxic.
- Endothelial cells subjected to nonfatal hypoxia undergo a constellation of phenotypic changes largely mediated by the early growth response-1 (egr-1) gene, which is activated via elk-1 under conditions of reduced oxygen tension. These phenotypic changes include signals that attract leukocytes and platelets and initiate local coagulation largely through the activation of tissue factor [5, 11, 24–27].
- If and when intermittent, discontinuous flow is restored, even transiently, the normal valve cycle [13, 14] resumes. The stagnant (hypoxemic) blood is evacuated from the valve pocket and replaced by fresh venous blood, containing living, active leukocytes and platelets. Phagocytes and platelets then marginate on and attack the necrotic parietalis endothelium of the lately hypoxic valve cusp leaflet, and the resulting accumulation of functioning-then-dying leukocytes (and platelets) is accompanied, or quickly succeeded, by fibrinogenesis.
- The next (and subsequent) sufficiently sustained episodes of continuous flow—once again rendering the valve pockets hypoxemic—may kill the blood cells that entered such valve pocket(s) during the previous discontinuous flow interludes. Continued alternation of continuous flow with brief discontinuous episodes may therefore result in the layered accumulation of generations of dead cells interspersed with dense extensions of fibrin, attached to the necrotic parietalis endothelium of the valve cusp leaflet.
- If and when this “protothrombus” grows sufficiently large to protrude from the mouth of the valve pocket into the vein lumen, it presents an abnormal surface on which the passing venous blood will coagulate, leading to further growth and generation of a frank and manifestly classically “layered” thrombus.
- Such a protruding, growing thrombus is subject to pressure from the flowing venous blood, and because it is “anchored” only to the necrotic (and hence fragile) parietalis endothelium, it is likely to embolize.

## 3. Some Comments on the Etiological Mechanism

We will describe our experimental confirmation of this account of the etiology of DVT at the beginning of Section 4.

The VCH thesis treats DVT as a consequence of vascular pathophysiology, not as a hematological disorder, emphasizing macroscopic processes rather than events at the molecular level. In other words, it is rooted in the classical literature. For instance, Virchow [28, 29] showed that venous thrombi form in valves, including the predominantly monocuspid “ostial” valves [20] at vein junctions [29], and he subsequently detailed the morphological distinctions between venous thrombi and blood clots [30], which are familiar to everyone who has seen a thrombus removed during venectomy or a pulmonary embolism removed during postmortem examination. These morphological distinctions (the massive accumulation of white cells and the very dense fibrin deposit in the oldest part of the thrombus, and the characteristic striations subsequently called the lines of Zahn) were later confirmed by others [31–33], and so was the finding that thrombi form on valves [34, 35], specifically on the inner faces of the valve cusps [23]. An autopsy study of leg vein thromboses revealed that most of the small (presumably recent) thrombi remained seated in the valve pockets, though some older thrombi were “free-floating”, probably as a result of dehiscence from their fragile moorings on the parietalis endothelium [36]. The VCH thesis predicted and explains all these observations [10, 11]. The autopsy study also showed that venous thrombi tended to form in the more distal parts of the limbs before the more proximal parts [36]. We may conjecture that a thrombus impairs discontinuity of flow in portions of the vein cephalad, increasing the likelihood of further thrombogenesis.

 It is well known that DVT and its sequelae can occur in a wide variety of circumstances in medical patients, surgical patients, and others. Indeed, many “risk factors” have been identified [37] and have led some authorities in the field to suppose that DVT is “multicausal” or “multifactorial” [38, 39]. Irrespective of circumstances, however, there is every reason to suppose that the thrombogenic process is always the same, as described by the VCH; for instance, all venous thrombi (and emboli resulting from the metastasis of venous thrombi) show the morphological features described by Welch [32] and others, suggesting a common pathway of formation.

Among the “risk factors” that have attracted particular attention during the past few years, various hereditary and acquired thrombophilias predominate. They have been extensively reviewed [40–43] and all are supposed to increase the likelihood of clinically significant DVT and PE. According to the VCH thesis (which is not inconsistent with the postulate that a venous thrombus grows more rapidly in a thrombophilic patient than in one with a supposedly normal coagulation mechanism), the patient is not necessarily at greater risk of DVT than normal individuals: thrombophilias are unlikely to affect the risk of *thrombogenesis*, though they could affect the *rate of thrombus growth and the probability of embolism*, and this might be thought to make their identification important for the management of patients.

Since thrombus *growth *involves the persistent deposition and accretion of blood elements via coagulation, anticoagulants are presumed by some authorities to inhibit it, though others prefer mechanical devices for prophylaxis, oxygenating the valve pockets by ensuring discontinuous blood flow [44–46]. However, it is not clear that anticoagulants prevent thrombus *initiation*. On the other hand, mechanical prophylactic methodologies, if they ensure regular emptying and refilling of the valve pockets, should strongly inhibit thrombogenesis according to the VCH thesis.

We will extend these remarks in the following section. However, we do not equate “*regular* emptying and refilling” with “*frequent* emptying and refilling.” According to the VCH thesis, the blood flow rate through the vein lumen is irrelevant to thrombogenesis; therefore, the use of mechanical devices to increase the laminar venous blood flow rate is fundamentally misguided, and the concept that “general venous stasis” is responsible for thrombogenesis encapsulates only part of the truth. In principle, injurious hypoxemia may occur in valve pockets *irrespective of the flow rate* whenever circulation is continuous (i.e., streamline). Therefore, the purpose of all mechanical approaches to prophylaxis should not be simply to accelerate venous return but to ensure emptying and refilling of the valve pockets at intervals of not more than 30–60 minutes, which should be sufficient to preclude hypoxic injury to the endothelial cells [11, 12, 18].

## 4. A Proposed Experimental Protocol for Evaluating Prophylactic Measures

The VCH thesis was confirmed experimentally in 1984 by experiments on anesthetized dogs [12]. A veterinary anesthetist ensured that the leg muscles did not contract during a prolonged period of immobility, so blood flow in the leg veins was continuous (streamline). As the (barbiturate) anesthesia lightened, the leg muscles underwent spontaneous “clonus”—very rapid convulsive movements—which restored discontinuous flow. A further extended period of immobility was then achieved by a second intravenous anesthetic dose, with recurrence of the clonic leg movements when the animals required a further injection. Subsequent histological examination of the leg veins showed several thrombi anchored in the valve pockets. Two points about this experimental production of venous thrombi must be emphasized: (1) the thrombi were induced by a noninvasive procedure that caused no overt injury to the veins but was confined to ensuring that several hours of continuous flow were interrupted by brief (less than 5 minutes) episodes of discontinuous flow; (2) they were morphologically indistinguishable from those observed clinically, that is, they corresponded to Virchow's [30], Welch's [32], and Aschoff's [33] descriptions of venous thrombi and were therefore not “blood clots” as defined by Virchow. Control animals, which received further doses of barbiturate to sustain the anesthesia and prevent discontinuous flow episodes, showed no thrombi and their valve pockets were unaltered.

This experimental protocol could readily be adapted to the purpose of testing the efficacy of prophylaxis. In the simplest case, two groups of dogs or other suitable animals would be required, and since the objective is to obtain a yes or no answer to the question “Does this approach to prophylaxis prevent DVT?” the groups would not need to be large. Both groups would receive anesthetic and muscle relaxant as in [12], ensuring protracted continuous flow in the leg veins punctuated by brief discontinuous flow episodes; one group would not be treated but the other would receive the selected putative prophylaxis. Histological comparison of the leg veins of the animals at the end of the experiment should provide the data to answer the question.

Mechanical prophylaxis could also be assessed by this procedure. Intermittent pneumatic compression at a lower frequency (once or twice an hour), and with less compression than is currently used, would expel blood trapped in motionless VVP, thus controlling the time for which such trapping continues unrelieved and obviating potentially injurious VVP hypoxemia. Were that to prove effective in all cases, as the VCH thesis predicts, it would be sensible to proceed to clinical trials. Such less-frequent artificial pulses would be more tolerable for patients than the disturbingly frequent ones currently used during intermittent pneumatic compression.

The other approach, previously proposed in [11], is 5–10-degree Trendelenburg/anti-Trendelenberg tilting of the animal, once again at (provisionally) 30- or 60-minute intervals, which should be sufficient to empty the leg vein valve pockets by gravity and allow them to refill with fresh blood before hypoxic injury to the endothelium can occur, notwithstanding the continuing continuity of flow like that during “relaxant” (curare-form) anesthesia. Once again, should that prove successful, it would be sensible to proceed to clinical trials. Slight, infrequent, end-to-end tilting of the bed should presumably be tolerable for most bed-bound patients, and the procedure would be unlikely to have discomfiting or adverse side effects.

If the proposed experimental protocol is used to evaluate the efficacy of an anticoagulant, different doses of the drug can be tried with a view to establishing the optimum. This approach to testing anticoagulants would have obvious clinical value. It would also lead to further testing and possible refinement of the VCH thesis, which implies (though this is not stated explicitly in [10, 11]) that histological examination of the leg veins of anticoagulant-treated animals at the end of the experiment would reveal prothrombotic nidi on the parietalis surfaces of venous valve cusps even if no overt (macroscopically visible) thrombus were present. Such nidi should, according to the thesis, consist mostly of white cells. As stated in Section 3, it is implicit in the VCH thesis that anticoagulants do not prevent the initiation of thrombogenesis, but they are likely to retard or prevent thrombus growth.

## 5. Conclusions

Recent publications [4, 6, 8] have raised doubts about anticoagulant prophylaxis against DVT and its sequelae, but this still remains the standard of care, with mechanical prophylaxis assigned a subordinate role. The VCH thesis would reverse this hierarchy. Rational approaches to mechanical prophylaxis, designed to ensure regular emptying and refilling of the venous valve pockets, should preclude thrombogenesis. Conversely, anticoagulants, though not precluding thrombogenesis, perhaps inhibit thrombus growth after pro-thrombotic nidi have formed.

Anticoagulant prophylaxis is not necessarily undesirable, though the risk of major bleeding—its best-known side effect—is of practical concern [6, 7]. The objective of any prophylactic measure should be to prevent the formation of thrombi that can lead to locally deleterious effects (e.g., postthrombotic syndrome) or to potentially fatal embolization. A method that inhibits thrombus growth could meet that objective as efficiently as one that prevents thrombus formation. Nevertheless, the prevention of thrombogenesis seems an inherently more satisfactory approach to prophylaxis than inhibition of growth, provided the chosen method has no known significant side effects.

The experimental protocol proposed here for evaluating both pharmacological (anticoagulant) and mechanical approaches to DVT prophylaxis would be simple and inexpensive and would curtail the time needed to collect sufficient patient data for valid statistical analysis. It should provide information justifying (or otherwise) the use of existing prophylactic measures and establish a basis for clinical trials of new approaches, leading to further elaboration of the VCH thesis and *ipso facto *new insights into the etiology of DVT.

There are obvious limitations to these proposals. Extrapolation from experimental animal data to clinical management of human patients is conjectural, so careful clinical trials would be required in the aftermath of the experiments. Animal experiments entail ethical concerns, which would need to be addressed. Furthermore, some approaches to prophylaxis, such as the proposed use of vegan or Mediterranean diets [6], could not be tested using this approach—large patient cohorts would still be necessary. Nevertheless, a method that reliably induces clinically accurate venous thrombi in animals provides a sound basis for testing most actual or possible approaches to prophylaxis, and because it would be rapid and would not require retrospective analysis of patient data, it seems reasonable to expect that it would result in a significant reduction in the incidence of DVT-related mortality and morbidity.
